# Innovative Approaches to NAFLD: Exploring the Role of Nicotinamide in Multicellular Microtissue Models of Liver Fibrosis

**DOI:** 10.1111/jcmm.70606

**Published:** 2025-06-04

**Authors:** Farnaz Sani, Shima Parsa, Kimia Falamarzi, Mohammadhossein Khorraminejad‐Shirazi, Negar Azarpira, Mahsa Sani

**Affiliations:** ^1^ Shiraz Institute for Stem Cell & Regenerative Medicine Shiraz University of Medical Sciences Shiraz Iran; ^2^ Wake Forest Institute for Regenerative Medicine Wake Forest School of Medicine Winston Salem North Carolina USA; ^3^ Zoonoses Research Center Jahrom University of Medical Sciences Jahrom Iran; ^4^ Department of Pathology, School of Medicine Jahrom University of Medical Sciences Jahrom Iran; ^5^ Department of Pathology, School of Medicine Shiraz University of Medical Sciences Shiraz Iran; ^6^ Student Research Committee Shiraz University of Medical Sciences Shiraz Iran; ^7^ Department of Tissue Engineering and Applied Cell Sciences, School of Advanced Medical Sciences and Technologies Shiraz University of Medical Sciences Shiraz Iran

**Keywords:** liver fibrosis, microtissue, NAFLD, NASH, nicotinamide, TGF‐β1

## Abstract

In non‐alcoholic fatty liver disease (NAFLD), characterised by progressive liver damage, inflammation, fibrosis and potential cirrhosis, treatment options are limited, with liver transplantation as the only definitive solution. To address this urgent need, in vitro and tissue engineering studies have explored new drugs. This study investigated the anti‐inflammatory and anti‐fibrotic potential of Nicotinamide, utilising promising preliminary data for treating NAFLD. Multicellular liver microtissues comprising LX2 stellate cells, HepG2 hepatocytes and HUVECs were generated to establish a robust preclinical model for fibrosis. Cell viability and histological assessments confirmed successful co‐aggregation within the microtissues. To induce fibrosis, they were treated with a palmitic and oleic acid mixture, followed by exposure to Nicotinamide. Treatment effectiveness was evaluated by analysing inflammatory factors, including transforming growth factor β1 (TGF‐β1), tumour necrosis factor‐alpha (TNF‐α) and interleukin 1 beta and interleukin 6. Extracellular matrix deposition was assessed by measuring collagen type I (COL I) and α‐smooth muscle actin (α‐SMA). Our data suggested that Nicotinamide application led to significant improvements across several measures. Specifically, it effectively reduced inflammatory response by reducing TGF‐β1 levels and decreasing COL I and α‐SMA levels. Furthermore, Nicotinamide was crucial in reducing reactive oxygen species levels, indicating that activation of HSC, inflammatory signals and oxidative stress work together. These in vitro findings suggest Nicotinamide may have therapeutic potential warranting further investigation in advanced preclinical models. Our findings demonstrated significant improvements across various parameters, including reduced expression of pro‐inflammatory cytokines and attenuated oxidative stress, underscoring the therapeutic potential of Nicotinamide in clinical studies.

## 1 Introduction

Fibrosis is identified by the excessive generation of extracellular matrix (ECM) in an organ after an injury or stimulus. Through the organ fibrosis process, a persistent injury and accumulating ECM in damaged parenchyma (remodelling process) may eventually result in organ failure [1]. Non‐alcoholic fatty liver disease (NAFLD), a harmful condition involving liver fibrosis, can be caused by prolonged high‐fat diet intake. The over‐accumulation of fat in the liver (known as steatosis) leads to the liver releasing pro‐inflammatory cytokines, among which TGF‐β1 plays a significant role. TGF‐β1 can promote the activation of hepatic stellate cells (HSCs) that lead to extracellular matrix production (ECM), which further progresses the NAFLD condition [2, 3].

At this level, fibrosis‐associated proteins such as collagen are going to be deposited, causing scar formation and cirrhosis in the liver [4, 5]. With all these consequences, NAFLD is a highly prevalent chronic liver disease with no definite treatment introduced yet [6]. At the end stages of the disease, cirrhosis cases would end up with surgical operations and liver transplant as the only available therapeutic option [7, 8]. Therefore, properly studying the condition has become an urge.

Animal models have been employed over the years to investigate NAFLD pathologically. However, they failed to remodel human NAFLD [9, 10] completely. Moreover, the recent FDA statement that novel therapies no longer require animal testing represents yet another big milestone. This action might be a crucial first step in the eventual abolition of animal testing after years of medication safety regulations [9]. Therefore, in vitro human fatty liver models in both 2D and complex 3D cultures have been introduced [10, 11]. As each cell type plays specific functions in the pathological phase of NAFLD, it is important to have both representing parenchymal and non‐parenchymal hepatic cells (HSCs and endothelial cells) to develop a proper in vitro human NAFLD model. Consequently, a 3D organoid type/spheroid developed by combining hepatic parenchymal cells (HepG2s), endothelial cells (HUVECs) and hepatic stellate cells (LX2s) would be an optimal choice to study various pharmacological options such as Nicotinamide (NA) on the NAFLD condition.

Nicotinamide (NA), as a form of niacin and a substrate of the enzyme NA N‐methyltransferase, has been reported to be among the promising therapeutic choices for improving liver injuries [11, 12]. NA is quickly absorbed from the small intestine when it's consumed orally [12, 13]. Previous studies on animal models demonstrated that NA intensely stopped DNA hypermethylation and recovered regular gene expression in fatty acid metabolism, oxidative stress, inflammation, cell proliferation and apoptosis. Regarding it's protective effect against the liver fibrotic process, Nicotinamide riboside proved to play a protective role in LX‐2 activation and the inflammation process in liver fibrosis by regulating the Smads signaling pathway acetylation [14]. The TGF‐β/Smad signalling pathway has been shown to significantly play a role in liver fibrosis pathogenesis and progression, and TGF‐β is considered an important cytokine in fibrosis progression and TGF‐β signalling blockage is confirmed to alleviate renal fibrosis in previously studied animal models [15, 16]. However, the detailed role of NA on human fatty liver improvement has yet to be thoroughly studied. In this study, taking advantage of a hepatic fibrosis model composed of the three mentioned human hepatic cell types, we aimed to evaluate the effect of NA administration on NAFLD progression, inflammation, oxidative stress and ECM accumulation.

## 2 Materials and Methods

### 2.1 Cell Lines and Cell Culture Conditions

The included cell types, involving HepG2, Lx2 and HUVECs, were purchased and provided by Pasture Institute (Iran, Tehran). Human umbilical vein endothelial cells (HUVECs), as primary cells isolated from the vein of the umbilical cord, were cultured in Dulbecco's Modified Eagle Medium/Nutrient Mixture F‐12 (DMEM/F12; Gibco, USA). HepG2 and Lx2 were routinely cultured in Roswell Park Memorial Institute (RPMI) 1640 Medium (Gibco, USA). The medium was supplemented with Penicillin–Streptomycin (100 U/mL) (Bioidea, Iran) with 10% (vol/vol) fetal bovine serum (FBS) (Gibco, USA) and 1× Glutamax (Bioidea, Iran), and incubated in a humidified atmosphere of 5% CO_2_ at 37°C. At about 70%–80% confluency, the cells were trypsinized by 0.05% trypsin/EDTA (Shellmax, China) and prepared for the experiment.

### 2.2 Multicellular Liver Microtissue (MLM) Preparation

The three cell types were set up in the MLMs at these ratios: HepG2 60%/Lx2 20%/HUVEC 20%. The process involved adding a cell suspension containing 5000 cells to each well from 96‐well round bottom ultra‐low attachment (ULA) plates. In 4 days, the cells naturally formed microtissue structures through self‐aggregation [17].

### 2.3 FFAs Supplement Was Used to Induce Fibrosis

MLMs were treated with a solution containing palmitic acid (PA) and oleic acid (OA) a day after cell seeding to cause liver fibrosis. To help with fatty acid intake in the cells, this solution (PA: 1/OA: 2, 500 M diluted in ethanol) was conjugated to 10% bovine serum albumin (BSA). On the following day, the mixture was diluted with a complete medium at a proportion of 1:10 and changed every other day [17, 18].

### 2.4 Treatments

Three experimental groups of liver microtissues were established: (1) untreated MLMs (Health group), (2) MLMs treated with FFAs alone (FFA group) and (3) MLMs treated with FFAs and 24 mM Nicotinamide (NA group).

### 2.5 Oil Red O (ORO) Staining

The existence of lipid accumulation in MLMs' cells was evaluated and confirmed using oil red O. Shortly, the MLMs were rinsed with PBS and then fixed in 4% paraformaldehyde solution for a duration of 15 min in each well. ORO working mixture was used to stain the MLMs for 20 min at room temperature. MLMs were then washed with PBS and examined under a microscope. Isopropanol was used to dissolve oil droplets for 20 min, and a plate reader (FLUOstar Omega, BMG Labtech) was used to detect absorbance at 518 nm.

### 2.6 Annexin V Flow Cytometric Analysis

The cellular apoptosis was evaluated using the Annexin V‐FITC apoptosis detection kit (Abcam) following the instructions provided by the manufacturer. In summary, the MLMs were washed with serum‐free media and then collected after centrifugation plus 5 min of incubation in Annexin V‐FITC. The bindings of Annexin V‐FITC were analysed with flow cytometry and a FITC laser detector (EX = 488 nm; Becton Dickinson, NJ). The obtained data were analysed using FlowJo Software [17].

### 2.7 Reactive Oxygen Species (ROS) Evaluation

The quantity of intracellular ROS generation was quantified using the assay kit specifically designed for detecting ROS (Abcam). The MLMs were rinsed with PBS and then exposed to 2′,7′‐dichlorofluorescein diacetate (DCFDA) in a 10% supplemented 1× buffer, incubating for 30 min at 37°C in a dark environment. During the incubation, cytoplasmic ROS converted DCFDA into a highly fluorescent compound called 2′,7′‐dichlorofluorescein (DCF). The fluorescence intensity was detected using a microplate reader (EX = 488 nm, EM = 535 nm) (FLUOstar Omega, BMG Labtech, Germany). By comparing the fluorescence intensity, determining and comparing the rate of ROS generation among the different groups was possible.

### 2.8 Determining Cell Proliferation and Viability

To investigate the proliferation of MLMs, the 3‐(4,5 dimethyl‐2‐thiazolyl)‐2,5‐diphenyl tetrazolium bromide (MTT) assay (M5655; Sigma‐Aldrich) was employed. In this assay, 200 μL of a 0.5 mg/mL MTT solution was added to each well, followed by a 4‐h incubation at 37°C. Then, the solution in each well was replaced with 100 μL of dimethyl sulfoxide (DMSO; Merck). The stained solutions' optical densities (ODs) were subsequently measured at 570 nm using a plate reader (FLUOstar Omega, BMG Labtech).

In order to assess the viability of cells in MLMs, the LIVE/DEAD assay utilising fluorescent dyes was employed. In this assay, a solution of 5 mg/mL fluorescein diacetate (FDA) was used to stain live cells, whereas a solution of 2 mg/mL propidium iodide (PI) was used to stain dead cells. The staining solutions were prepared in a culture medium without FBS. Subsequently, the MLMs were incubated with the staining solutions at room temperature for 5 min in a dark environment. The samples were analysed using fluorescent microscopy to visualise and differentiate between live and dead cells.

### 2.9 Protein Expression

The MLMs were fixed in a 4% paraformaldehyde solution for fixation and then rinsed with PBS to remove any excess fixative. The next day after treating groups with anti‐αSMA and CD31 antibody overnight at 4°C (dilution 1:50; Abcam), a secondary antibody (dilution 1:200; Abcam) was used. Afterward, the MLMs were subjected to protein detection using horseradish peroxidase (HRP) conjugated antibody at a dilution of 1:10,000 (Abcam) and the DAB+ chromogen‐substrate system at a dilution of 1:50 (Dako). As a negative control, the same procedure was followed without the primary antibody incubation.

The immunofluorescence technique assessed COL 1A1 expression. Following the fixation step, the MLMs were incubated with primary antibodies against COL I at a dilution of 1:200 (Abcam) and incubated for 1 h at room temperature in a PBS solution containing 0.1% BSA, 1% goat serum and 0.05% Tween 20. MLM samples were washed with PBS to remove any unbound primary antibodies, and a fluorescent secondary antibody was applied. Hoechst stain was used and incubated for 5 min to evaluate the nuclei. The samples were then observed under fluorescence microscopy to check the fluorescence signals of the labelled antibodies and the stained nuclei.

### 2.10 Enzyme‐Linked Immunosorbent Assay (ELISA)

The production levels of tumour necrosis factor‐alpha (TNF‐α), interleukin 1 beta (IL‐1β) and interleukin 6 (IL‐6) were assessed using the ELISA technique. The conditioned media from the 3D samples were collected and preserved at −70°C for further analysis. The quantification of secreted TNF‐α and IL‐1β (both from Eastbiopharm, China), as well as IL‐6 (Diaclone, France), was performed based on the instructions provided by the respective manufacturers.

### 2.11 Levels of Liver Enzymes

The supernatant of each well in the MLMs was collected and centrifuged at 3000 × *g* for 10 min. The obtained supernatant was used to measure the levels of alanine aminotransferase (ALT), aspartate aminotransferase (AST), albumin (ALB) and urea using a Biosystems kit.

### 2.12 RNA Extraction and Quantitative Real‐Time Polymerase Chain Reaction

To quantify the mRNA levels of collagen type I (COL1A1, NM_000088), Transforming growth factor β1 (TGF‐β1, XM_011527242.3) and α‐smooth muscle actin (α‐SMA, NM_001406462.1), quantitative reverse transcription real‐time polymerase chain reaction (qRT‐PCR) was performed. The specific primers for each target gene were applied with reverse transcription and PCR amplification. The mRNA levels were assessed by a real‐time PCR instrument and analysed based on the cycle threshold (*C*_t_) values.

Extraction of the total RNA from the 3D model was completed by an RNeasy Plus Mini Kit (QIAGEN). Complementary DNA (cDNA) was synthesised using the RevertAid H Minus First strand cDNA synthesis kit (Thermo Scientific, USA) following the instructions provided by the manufacturer. Until further analysis, the cDNA aliquots were stored at −20°C. To assess the relative gene expression, real‐time reverse transcription polymerase chain reaction (RT‐PCR) was performed using the SYBR Premix Ex TaqTM II kit (Takara, Japan) on an Applied Biosystems StepOnePlus System (ABI, USA). In order to normalise, the reference gene GAPDH was used. Also, the fold change expression of each gene was determined using the $2^{-\Delta \Delta C_{t}}$ method.

### 2.13 Data Analysis

A minimum of three repetitions was used to gather all the data. Standard deviation is displayed as error bars. One‐way analysis of variance (ANOVA, Tukey HSD as a post hoc test) was used for statistical analyses to check multiple assessments. A *p* value of 0.05 or less was considered statistically significant.

## 3 Results

### 3.1 Development and Viability of the MMLs

**FIGURE 1:**
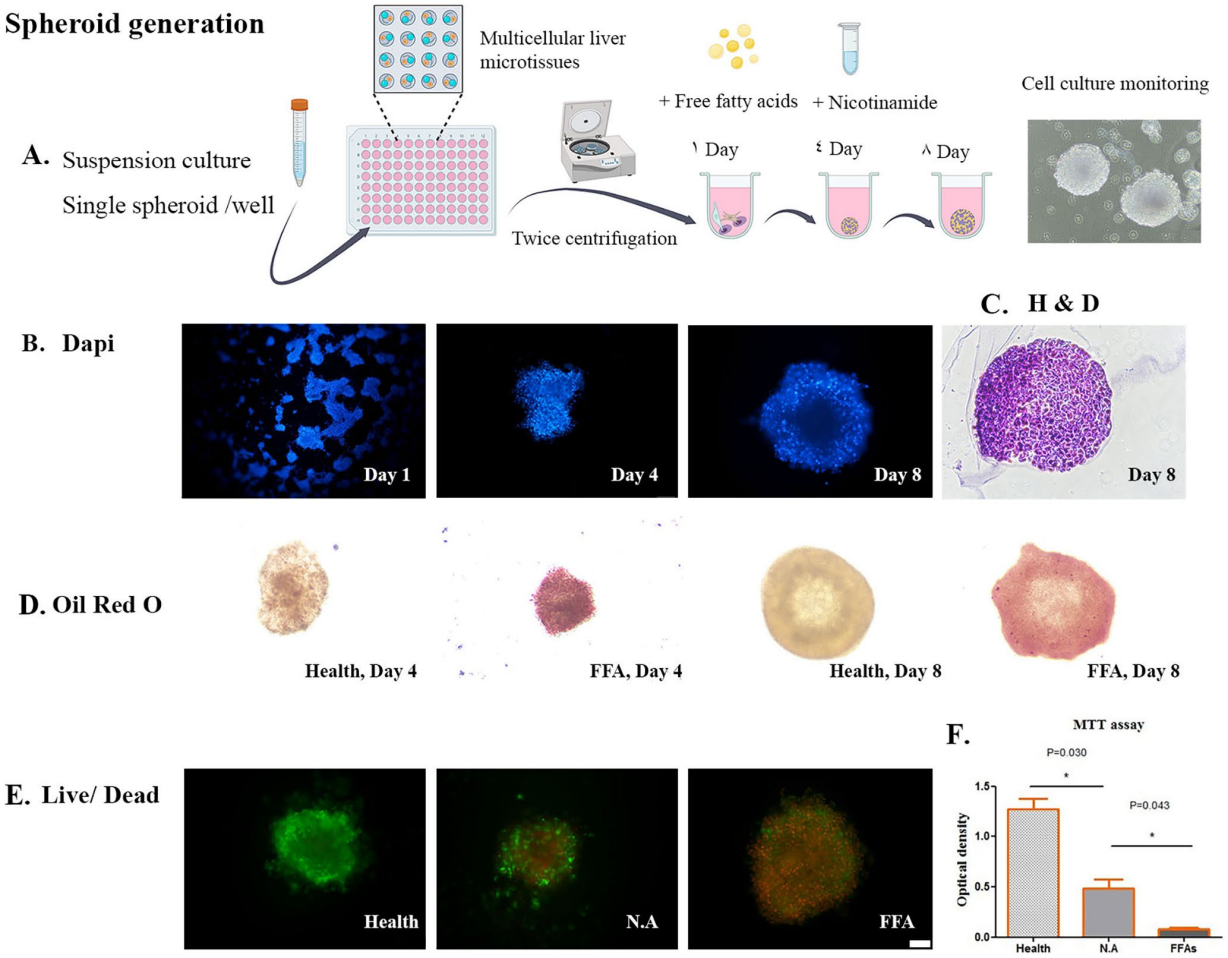
(A) Schematic illustrations of the formation and development of multicellular liver microtissues (MLMs) on different days; nicotinamide as treatment was added on Day 4. (B) Fluorescence images of MLMs that were composed of parenchymal and non‐parenchymal cells. (C) haematoxylin and eosin staining on Day 8. (D) Accumulated lipid droplets on Days 4 and 8. (E) Live (green)/dead (red) assay and (F) cell proliferative capacity of the treated MLMs were determined by MTT assay on Day 8 (*p < 0.05).

As described before, the fatty liver samples were set up and characterised. An oil red O staining assay was performed to measure lipid accumulation qualitatively (Figure 1D). To qualitatively confirm the MML's viability, LIVE/DEAD and the MTT assays were applied. Even with medium replacement every other day, after 8 days of cell culture, the number of dead cells (red dots) visible under a fluorescence microscope in the negative controls was noticeably more remarkable than in the positive controls. Interestingly, the live/dead outcomes in the NA group were more comparable to the control group (Figure 1E).

To further quantify the influence of NA on the MMLs, their proliferation was evaluated using the MTT assay (Figure 1F). The results from the MTT assay further confirmed the observation that after 8 days of cultivation, NA significantly increased cell proliferation at the absorbance of 570 nm.

### 3.2 Nicotinamide Improves NAFLD‐Induced Increased Reactive Oxygen Species (ROS)

**FIGURE 2:**
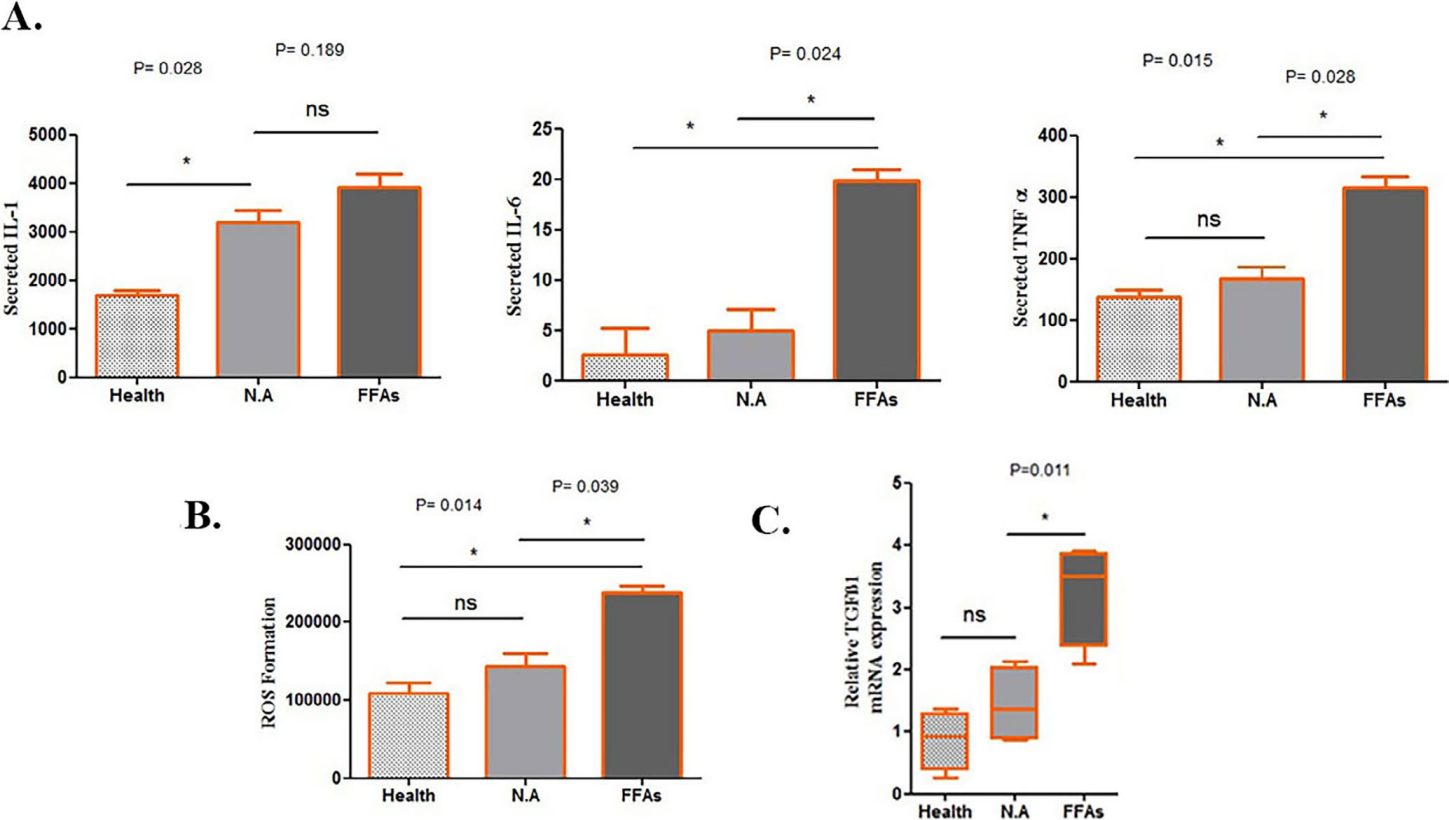
(A) interleukin‐1 beta (IL‐1β), interleukin 6 (IL‐6), and tumour necroes factor alpha (TNF‐α) production with ELISA assay after treatments. (B) Reactive oxygen species (ROS) measurements. (C) Transforming growth factor beta (TGF‐β) gene expression analysis. Stars indicate a statistical difference (*p < 0.05).

Oxidative stress, defined by overexpression of ROS (56,57), is one of the major complications in fatty liver development (17). To investigate oxidative stress, levels of ROS have been experimented with in both the control and NA‐treated groups through the ROS assay kit. Based on the findings of this study, NA could significantly decrease the expression of ROS, and the levels of generated ROS had no significant differences compared to the health group (Figure 2B).

### 3.3 Pro‐Inflammatory Cytokines Profiling Upon Nicotinamide Administration

The pro‐inflammatory cytokines, including early‐phase pro‐inflammatory cytokines (IL‐1), late‐phase pro‐inflammatory cytokines (IL‐6), tumour necrosis factor‐alpha (TNF‐α) and TGF‐β1 have been reported to be upregulated through the fatty liver condition. To investigate the NA application of the MLM models, the levels of these factors have been checked through either ELISA or QR‐PCR experiments (Figure 2A,C). The obtained data showed that applying NA resulted in a reduction in the expression of early‐phase pro‐inflammatory cytokines, IL‐1, with a significant reduction in the expression of late‐phase pro‐inflammatory cytokines, IL‐6 (*p* value = 0.024), TNF‐α (*p* value = 0.028) and TGF‐β1 (*p* value = 0.011). A similar study by our team has also reported a similar reduction in lipid accumulation as well as alleviating the increased pro‐inflammatory cytokines upon administration of mesenchymal stem cells‐derived exosomes [17].

### 3.4 Nicotinamide Application Reduced the Extracellular Matrix (ECM) Over Expression

**FIGURE 3:**
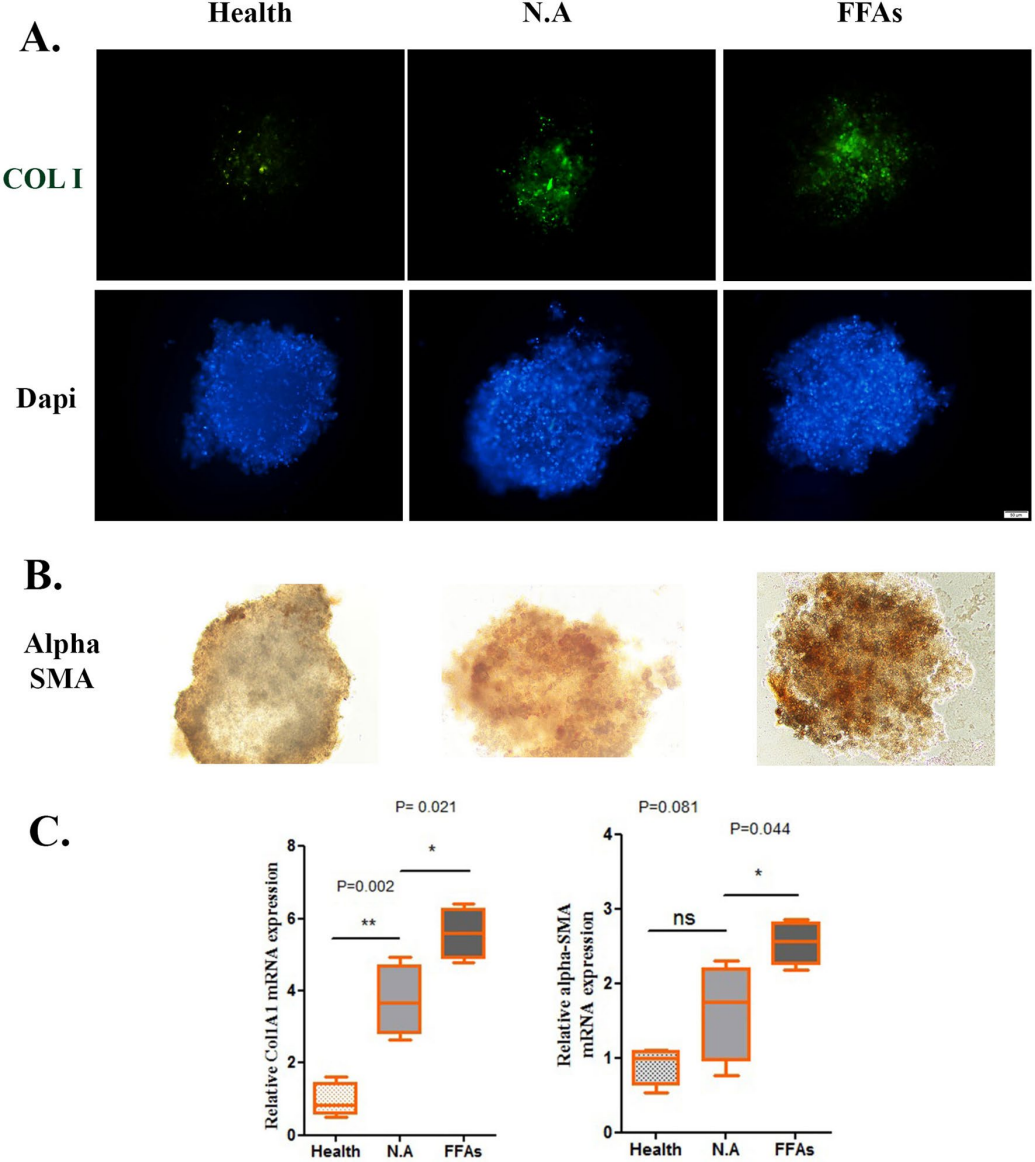
(A) Representative fluorescence images (scale bars: 50 μm). (B) The expression level of α‐smooth muscle actin (α‐SMA) in multicellular liver microtissues at different study groups. (C) Gene expression of collagen I (COL I), and α‐smooth muscle actin (α‐SMA). Levels of gene expression were normalised against a control group (data are expressed as the mean ± standard error of the mean, *n* = 3 per group, *p < 0.05, and **p < 0.01).

One of the ways to diagnose non‐alcoholic fatty liver is based on over generation of fibrosis markers including Col I and αSMA. The excessive production of these factors could be decreased by administration of an effective substance against NAFLD. As a result, either protein expression experiments (to check Col I) or QRT‐PCR (to check αSMA) were used in this work to examine the levels of these elements. Here, it is demonstrated that upon application of NA, the levels of αSMA significantly decreased in a comparison with the negative control model (*p* value = 0.044). Also, αSMA expression levels have shown to be similar to the health groups. However, the Col I level illustrated significantly higher expression in the NA group in comparison to the health group (*p* value = 0.002) whereas it also has a significantly lower expression relative to the FFAs (negative control) groups (*p* value = 0.021) (Figure 3).

### 3.5 Liver Enzymes Profiling Through the Application of NA

**FIGURE 4:**
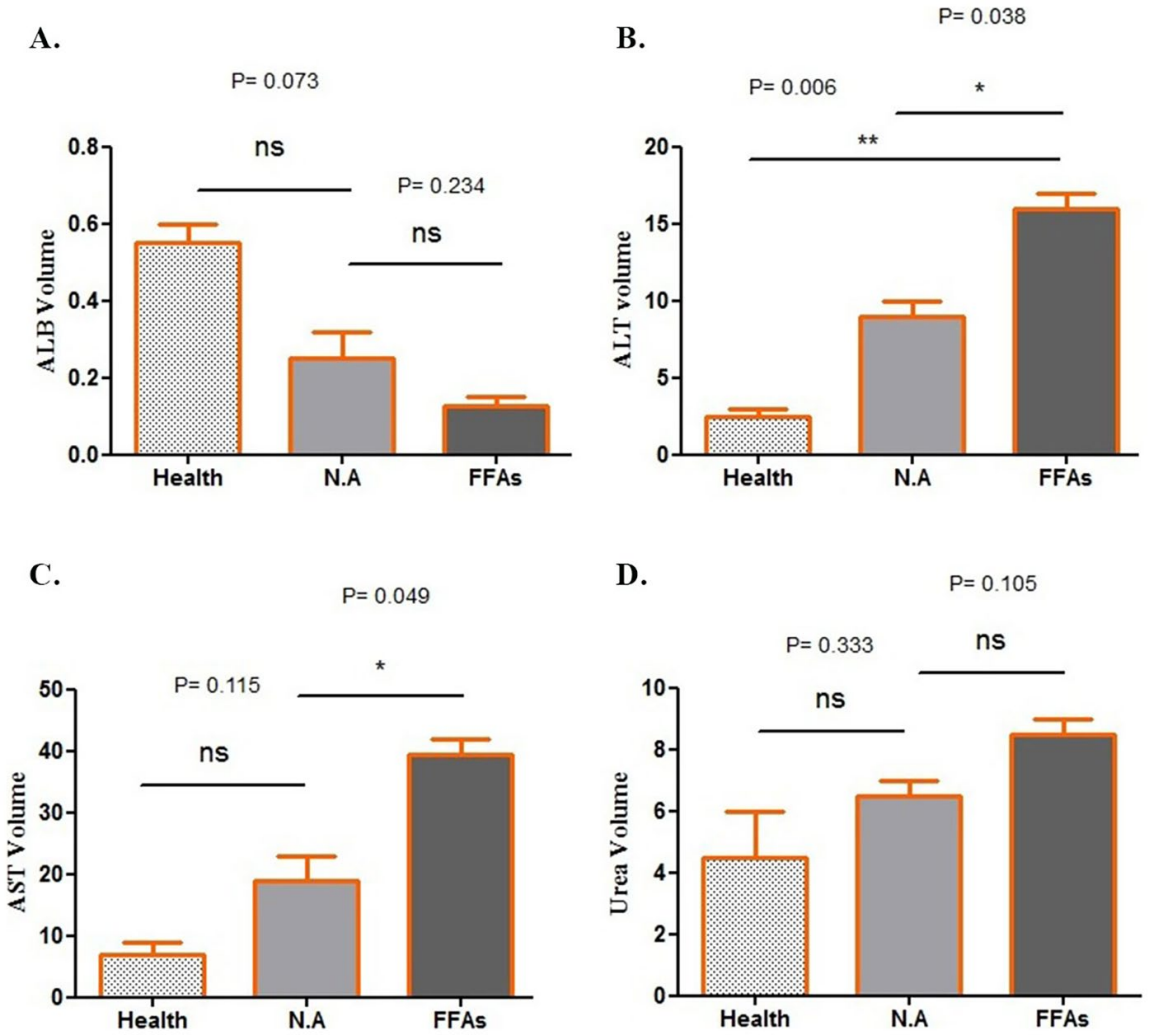
The levels of secreted (A) albumin (ALB), (B) alanine aminotransferase (ALT), (C) aspartate aminotransferase (AST), and (D) urea were measured by Biosystems Kits (*p < 0.05 and **p < 0.01).

Hepatic enzyme levels are frequently employed as biochemical indicators to assess liver damage (18). In this study, the related levels of AST, ALT and ALB were assessed by a Biosystems kit. The effects of NA on liver enzyme expression in all groups are shown in Figure 4. Both AST and ALT levels experienced a significant expression decrease relative to the FFAs group (*p* values, 0.49 and 0.38, respectively) whereas despite a reduction in its levels, the ALB generation did not show a significant difference relative to the FFAs. Also, When comparing urea levels between different groups, there were no significant differences.

## 4 Discussion

NAFLD is one of the worldwide severe conditions for which there is no absolute treatment or extensive study model. Although animal models have played crucial roles over the years to enhance the breadth of physiological understanding, the inherent genetic and epigenetic differences between various animals and humans have limited their adequacy in several drug discovery contexts. The failure rates of experimental drugs during clinical testing exceed 80%, with 60% attributed to efficacy and 30% to toxicity issues [19, 20]. Significant progress has been made in the development of alternative preclinical tools, which have amassed a wealth of robust data. These tools demonstrate a strong correlation with their potential for translation to clinical applications. This progress has led to a point where the United States Food and Drug Administration (FDA) no longer mandates animal testing as a prerequisite for seeking approval for human drug trials [21]. Despite the several applications of 2D models, they have not been capable of representing the complexity of the 3D natural tissues. Therefore, the transition from animal research to human studies is mostly anticipated to be bridged by 3D cell culture systems as promising in vitro strategies. In this study, a 3D model of a multicellular fatty liver using human hepatocytes has been suggested as an excellent model to explore how Nicotinamide affects the disease in all aspects of inflammation, oxidative stress and liver enzymes.

One of the main features of the announced spheroid models in this investigation is the presence of three hepatocytes in the same ratio as in natural human liver tissue (parenchymal 60% vs. non‐parenchymal 40%) [17, 22]. Previous reports indicated that although HepG2 spheroid cells were weakly attached and could not produce ECM components, co‐culturing HepG2 with LX2 improved the spheroids' density [23]. In another study, Lasli et al. [24] reported that NAFLD pathogenesis might be more accurately replicated when HUVECs (20%) were added to HepG2 spheroids. Therefore, confirmed by the outcomes of our work, a 3D model involving HepG2s, LX2s and HUVECs could be an optimum option to complete the previous studies.

Earlier established, the steatotic liver resulting from the buildup of fat in hepatocytes is vulnerable to secondary hits such as hepatic inflammation and fibrosis [25, 26]. In this regard, based on the results from this report, TGF‐β1 has its highest levels in the fatty liver models that are confirmed by previous reports [3, 27]. TGF‐β1 stimulates gene transcription of Col 1 a (I), one of the crucial genes encoding the polypeptides that make type 1 collagen. Type 1 collagen is the most prevalent byproduct of fibrosis and the key element in over‐secreted extracellular matrix from the activated HSCs during the fibrosis process [28]. Likewise, the ubiquitous transcription factor NF‐kB is essential for the development of hepatic fibrosis and the activation of HSC [29]. HSC activation is the main step in hepatic fibrosis resulting in high expression of ECM mainly containing COL I and α‐smooth muscle actin (α‐SMA). Confirmed by the data from this report, NA can significantly reduce TGF‐β1 levels leading to a decrease in both COL I and α‐SMA in the fatty liver models in this study. In line with this data, the ameliorative effect of NA on TGF‐β1 is previously reported by Zhang et al. [16] in their investigation on renal fibrosis. Moreover, NA has been suggested to play anti‐inflammatory roles in sepsis [30, 31] and microglial inflammation [32]. Its anti‐inflammatory effect is also confirmed by in vivo findings by Han et al. [33].

In the same way, the results of this investigation point to the possibility of hepatocytes' production of ROS induced by fat accumulation from palmitic acid and oleic acid induction. Following its administration to the NAFLD spheroid models, NA demonstrated a major protection against ROS accumulation. These data are in line with previous reports highlighting the role of NA against oxidative stress in pathological human epidermis and neurodegenerative disease [34, 35]. NA is a precursor of NAD biosynthesis, which is a key modulator of antioxidant defences [32, 33]. NAD complementation has proved to improve plasma lipid and cholesterol profiles, helping to attenuate metabolic disorders and hepatic steatosis [36]. NA has also been reported to suppress the NADPH oxidase, which is a critical enzyme in various cellular biosynthetic pathways and is responsible for the resulting generation of ROS [37]. Furthermore, NA plays a protective role against hepatic changes in the glucose‐6‐phosphate dehydrogenase (G6PD), which is crucial for producing NADPH and restoring intracellular redox state [35, 38, 39]. In short, it can be concluded that the anti‐oxidative potential of NA is probably through its support in maintaining NAD and NADPH levels. This further supports the claim that activation of HSC, inflammatory signals and oxidative stress show to work in concert [28]. Also, the incomplete restoration of ALB and urea levels suggests nicotinamide may need more time or combination therapies to fully reverse NAFLD. Although it effectively reduces fibrosis (TGF‐β1/COL I/α‐SMA), functional recovery appears delayed—matching clinical patterns where liver function recovers slower than structural improvements.

The findings of this work also highlighted that the alleviation of liver fibrosis factors after NA administration is in cooperation with an improvement in AST and ALT levels. In agreement with this data, previously reported by Shi et al. and Chen et al. [40, 41], NA was shown to be highly effective in reducing AST and ALT levels in liver injury induced by alcohol or acetaminophen abuse.

## 5 Conclusion

**FIGURE 5:**
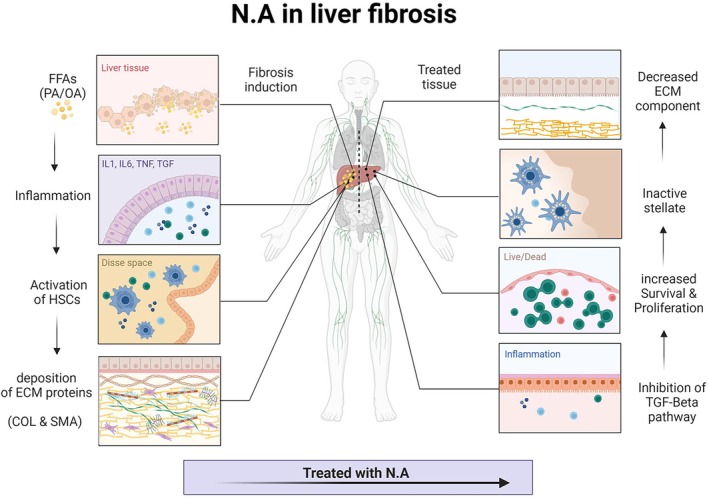
After fibrosis induction, the inflammatory response can activate stellate cells, leading to an increase in extracellular matrix (ECM) components. In this study, we employed nicotinamide as an inhibitor of the transforming growth factor beta (TGF‐β) pathway, which is known to be involved in fibrosis. By targeting this pathway, we aimed to prevent excessive cell proliferation and promote the restoration of tissue to a normal state.

Taken together, the fat accumulation in the spheroid models upregulates oxidative and inflammatory factors and increases the synthesis of collagen and α‐SMA. The fat accumulation‐mediated changes were mitigated by NA treatment, pointing out that it acts and exerts its anti‐fibrotic potential by down‐regulating TGF‐β1‐mediated production of collagen and antagonising ECM synthesis in liver microtissues (Figure 5). The present study also demonstrates that NA exerts antioxidant effects in NAFLD models by inhibiting cellular ROS accumulation. Notably, the anti‐inflammatory and antioxidant effects of NAM in NAFLD models are likely related to its activation of SIRT1 by de‐repressing SIRT1 expression and providing its activator, NAD+. Nonetheless, the present study suggests the potential of NA as a therapeutic agent for NAFLD conditions.

## Author Contributions

**Farnaz Sani:** data curation (equal), formal analysis (equal), investigation (equal), methodology (equal), validation (equal). **Shima Parsa:** data curation (equal), investigation (equal), writing – original draft (equal). **Kimia Falamarzi:** methodology (equal), project administration (equal), writing – original draft (equal). **Mohammadhossein Khorraminejad‐Shirazi:** formal analysis (equal), methodology (equal). **Negar Azarpira:** conceptualization (equal), data curation (equal), funding acquisition (equal), supervision (equal), writing – review and editing (equal). **Mahsa Sani:** conceptualization (equal), data curation (equal), formal analysis (equal), supervision (equal), validation (equal).

## Disclosure

Limitations of the study: In recent years, microfluidic devices have been utilised for applications like tissue engineering, diagnostics and drug screening. We suggest using microfluidic devices to improve the control in cultivation systems.

## Conflicts of Interest

The authors declare no conflicts of interest.

## Data Availability

All raw data used in this study can be acquired by contacting the corresponding authors, Dr. Negar Azarpira and Mahsa Sani.
